# Ethyl acetate extract of *Terminalia chebula* alleviates DSS-induced ulcerative colitis in C57BL/6 mice

**DOI:** 10.3389/fphar.2023.1229772

**Published:** 2023-12-12

**Authors:** Wan-Rong Dong, Yao-Yao Li, Tian-Tian Liu, Gao Zhou, Yu-Xin Chen

**Affiliations:** Key Laboratory of Fermentation Engineering (Ministry of Education), Cooperative Innovation Center of Industrial Fermentation (Ministry of Education and Hubei Province), School of Biological Engineering and Food, Hubei University of Technology, Wuhan, China

**Keywords:** *Terminalia chebula*, ulcerative colitis, inflammation, TLR4/MyD88/NF-κB, intestinal flora

## Abstract

**Background:** The Chinese pharmacopeia records *Terminalia chebula* as effective in treating prolonged diarrhea and dysentery, blood in the stool, and prolapse. Modern pharmacological research proves it has multiple pharmacological benefits, including antioxidant, anti-inflammatory, analgesic, hepatoprotective, neuroprotective, and other properties.

**Objectives:** This study aims to clarify the role of *Terminalia chebula*’s ethyl acetate extract (TCEA) on ulcerative colitis (UC) induced by dextran sodium sulfate (DSS) in mice, as well as explore the potential mechanism of action.

**Materials and methods:** The variation of different extracts of *T. chebula* was detected using the HPLC technique, and the main components in TCEA were identified. DSS was used to establish a mouse model to mimic the physiological state of UC in humans; the alleviating effect of TCEA and positive control 5-ASA on UC mice were evaluated by gavage treatment. Disease progression was assessed by monitoring the mouse’s weight change and disease activity index (DAI). The changes in colon tissue were estimated by measuring colon length, HE, and AB-PAS staining and detecting oxidative stress parameters. The results draw from Western blot and real-time PCR showed the TLR4/MyD88/NF-κB pathway may involve in the anti-inflammatory activity of TCEA. Furthermore, the gut flora sequencing technique was employed to monitor the differentiation of intestinal microbiota of mice induced by DSS and TCEA treatment.

**Results:** TCEA significantly lowered DAI scores and inhibited the weight loss and colonic shortening induced by DSS. The colon histomorphology and oxidative stress levels were enhanced after TCEA treatment compared with DSS induced UC group. TCEA attenuated the inflammatory response by regulating TLR4/MyD88/NF-κB pathway activation. Intestinal flora sequencing showed that DSS and TCEA greatly impacted mice’s composition and diversity of intestinal microorganisms. But TCEA increased the abundance of Bacteroidetes and decreased the abundance of Firmicutes and Proteobacteria compared with the DSS group, which contributed a lot to returning the intestinal flora to a balanced state.

**Conclusion:** This study confirms the alleviating effect of TCEA on UC and provides new ideas for developing TCEA into a new drug to treat UC.

## 1 Introduction

Inflammatory bowel disease (IBD) is a chronic inflammatory disease that damages the colon, with Crohn’s disease (CD) and ulcerative colitis (UC) being the main types. The main characteristic of UC is mucosal inflammation starting from the rectum and continuing to the colon. Its main symptom is bloody diarrhea, which can be diagnosed by colonoscopy and histological examination at diagnosis (Ungaro et al., 2017). The pathogenesis of UC is influenced by several factors, including genetics, epithelial barrier defects, immune response dysregulation, diet or environmental factors, and others (Ng et al., 2013; Aslam et al., 2022). UC is gradually evolving to be a global burden due to the incidence of it is rising in both developed and developing countries, along with UC causing a large increase in colorectal cancer (CRC) morbidity and mortality (Kobayashi et al., 2020; Olén et al., 2020). UC treatment aims to induce and maintain remission with the long-term goal of preventing disability, colectomy, and colorectal cancer (Ungaro et al., 2017). Currently, the drugs used to treat UC include 5-aminosalicylic acid drugs, steroids, and immunosuppressants, whereby 5-aminosalicylic acid drugs used for mild to moderate patients and immunosuppressants or biologic drugs used for moderate to severe patients (Ungaro et al., 2017; Aslam et al., 2022). However, up to 15% of patients require surgical treatment (Feuerstein et al., 2019). The existing treatment methods for UC are still unsatisfactory, mainly because they will cause diverse adverse reactions, such as drug resistance, vomiting, and alopecia (Rosenberg and Peppercorn, 2010; Renna et al., 2014). Due to a worldwide rise in the incidence of UC and the serious side effects that accompany most of the available treatments, physicians and pharmacists worldwide are looking for safer drugs with fewer side effects for UC. (Rosenberg and Peppercorn, 2010; Molodecky et al., 2012; Bressler et al., 2015). It has been reported that the occurrence of UC is closely related to the dysregulation of Nrf2/NLRP3, TLR4/My D88/NF-κB, JAK2/STAT3 and other signaling pathways (Liu and Jiang, 2020; Yang et al., 2021; Chao et al., 2022). TLR4 is an essential member of the pattern recognition receptor, which plays a necessary role in innate immunity. Activation of TLR4 leads to the transcription of genes for inflammation and subsequently participates in the development of UC by acting on MyD88 and NF-κB (Liu et al., 2022a). Previous studies have also shown that the TLR4/MyD88/NF-κB signaling pathway is abnormally activated in the intestinal mucosa of patients with UC (Yang et al., 2021).

Traditional Chinese medicine (TCM) is a system of medicine with a long history based on the theory and practice of Chinese culture. It has unique advantages in treating chronic diseases since its multi-level, multi-session, multi-target, and low side-effect properties (Liu et al., 2022b). TCM considers UC mainly caused by “stagnation of dampness and heat in the internal organs and imbalance of Qi and blood”. Doctors can employ a variety of remedies to achieve a therapeutic effect (Zhang et al., 2013; Yang et al., 2022). It has been shown that single herbal extracts, herbal pairs, classical herbal prescriptions, and effective components of herbal medicines are useful in the treatment of UC, mainly in protecting the integrity of the intestinal mucosal barrier, inhibiting the release of inflammatory factors, regulating oxidative stress and so on (Liu et al., 2022a). For instance, Chinese scholars have demonstrated that the Chinese herbal medicine *Schisandra chinensis* (Turcz.) Baill has obvious effects on DAI score and colon tissue damage in UC mice and can increase the levels of ZO-1 and occludin to maintain intestinal barrier function (Bian et al., 2022). Baicalein, the active ingredient in *Scutellaria baicalensis* Georgi, distinctly alleviated the sickness of DSS-induced UC mice mainly by enhancing the intestinal epithelial barrier through AhR/IL-22 pathway in ILC3s (Li et al., 2021). Also, TCM has been confirmed to influence the intestinal flora, including changing gut microbiota composition, regulating microbiota metabolites, improving the intestinal barrier, and others. For example, the classical Chinese medicine formula Huangqin decoction can restore the imbalance of intestinal microorganisms by promoting the growth of beneficial bacteria and decreasing the content of pathogenic bacteria (Feng et al., 2022).

*Terminalia chebula* is the dried ripe fruit of *T. chebula* Retz. And its variety *T. chebula* Retz. var. *tomentella* Kurt., distributed in Tibet, Yunnan, Guangxi, and other places in China (Li et al., 2019; Committee, 2020). Current Chinese Pharmacopoeia states that *T. chebula* belongs to the lung and large intestine meridians, tastes bitter, sour, astringent, and flat, and can be used for the treatment of prolonged diarrhea and dysentery, blood in the stool, and prolapse (Committee, 2020). Modern pharmacological researches have revealed that *T. chebula* contains tannins, flavonoids, and other compounds with various pharmacological functions, such as antioxidant, antitumor, analgesic, and so on. Korean scholars found that *T. chebula* water extract treatment resulted in improving the skin barrier, reducing immune cell infiltration, and inhibiting the elevation of inflammation-related mediators, with positive efficacy in atopic dermatitis in mice, suggesting *T. chebula* extract has an excellent anti-inflammatory effect (Kim et al., 2022). Moreover, it was discovered that the ethanolic extract of *T. chebula* was able to alleviate the symptoms of acetic acid-induced ulcerative colitis in rats, as shown by better crypt destruction and lower levels of myeloperoxidase in rat colon tissue (Gautam et al., 2013). *T. chebula* has appeared to have great promise in treating inflammatory diseases, but there are few studies on the ethyl acetate extract of *T. chebula* (TCEA) against DSS-induced UC. Based on these, we aim to prove that TCEA can reverse UC by using an animal model induced by DSS and to investigate biomarkers and intestinal flora altered by TCEA treatment after the rat’s colon suffers acute injury.

## 2 Materials and methods

### 2.1 Reagents

*T. chebula* fruits were purchased from the Hehua Chi Medicine Market in Chengdu, Sichuan Province, and the sample was preserved and identified by Dr. Gao Zhou from Hubei University of Technology (sample number: 20211213). Dextran Sulfate Sodium Salt (DSS) (MW: 36000∼50000) was purchased from MP Biomedicals company (Santa Ana, California, United States). 5-Aminosalicylic acid was purchased from Shanghai Yi’en Chemical Technology Co., Ltd. (Shanghai, China). CAT, GSH, MDA, GSH assay kits, and staining reagents were purchased from Beyotime Biotechnology (Shanghai, China). All analytical standards were purchased from Shanghai Yuanye Biotechnology Co., Ltd. (Shanghai, China). TLR4, β-actin antibody, and horseradish peroxidase-conjugated anti-rabbit IgG purchased from Servicebio Biotechnology Co., Ltd. (Wuhan, China). MyD88, NF-κBp65 antibody purchased from Proteintech Group (Wuhan, China). Methanol and acetonitrile were chromatographically pure from Thermo Fisher Scientific Inc. (Shanghai, China).

### 2.2 Experimental animals

Forty male SPF-grade C57BL/6 mice, weighing 20–25 g, were purchased from Hubei Provincial Center for Disease Control and Prevention. The animals received food and water freely at a room temperature of 23°C ± 2°C and a humidity of 55% ± 5% for 5 days of accommodation before the experiments. All experimental operations were carried out under the supervision of the IACUC (Institutional Animal Care and Use Committee) of the Hubei University of Technology, and experimental manipulations followed the relevant regulations.

### 2.3 Extraction and fractionation of TCEA

Crushed the fruits of *T. chebula* with a grinder, weighed 200 g powder, extracted three times with ten times volume of 95% ethanol by ultrasonication, and concentrated by rotary evaporation after the extraction. A certain amount of ethanolic extract of *T. chebula* be diluted using pure water and sequentially extracted with ten times the volume of petroleum ether, dichloromethane, ethyl acetate, and n-butanol, the extracted solutions concentrated by rotary evaporation and dried to obtain five extract fractions.

### 2.4 HPLC analysis of the active ingredients of TCEA

Using Thermo Fisher U3000 high-performance liquid chromatography system with a C^18^ column (COSMOSIL, 4.6 mm × 250 mm, 5 μm) was used to distinguish components from the total 95% ethanol extract and five fractions of *T. chebula* and then characterize the main active ingredients in TCEA by using six standard compounds. The injection volume was 10 μL, the flow rate was set to 1 mL/min, the column temperature was kept at 30°C, and the wavelength of the UV detector was set at 275 nm. The mobile phase comprised solvent A (0.1% formic acid in water) and solvent B (acetonitrile). The gradient elution was constant at 5% solvent B for 5 min, followed by a rise from 5% to 10% in 6 min, from 10% to 15% in 5 min, and kept at 15% for 4 min. In the next 9 min, solvent B rose to 18%, then continued to rise to 20%, maintained for 14 min, and finally, solvent B returned to 5% from 20% within 3 min, followed by a gradient equilibration at 5% for 4 min.

### 2.5 Experimental design

Forty C57BL/6 male mice were randomly divided into five groups with eight mice in each group: Control group; DSS group; 5-ASA group (200 mg/kg); TCEA low-dose group (100 mg/kg); and TCEA high-dose group (200 mg/kg). All groups, except the control group, were given 2.5% DSS freely for six consecutive days to build an acute UC model. The 5-ASA group and the TCEA-treated group were intervened according to the above doses by intragastric administration once daily. In contrast, the control and DSS groups were given the same volume of saline. On day 7, DSS was stopped provided in all groups, and saline, 5-ASA, and TCEA were continued to be administered to corresponding groups. On day 8, all mice were sacrificed by carbon dioxide (Figure 3A).

### 2.6 DAI assessment

From day 1 to day 8, relevant indices were recorded daily, including body weight, diet consumption, water consumption, fecal traits, and blood in the stool. The disease activity index (DAI) can calculate as follows: DAI = (weight change score + fecal trait score + blood in stool score)/3 (Cooper et al., 1993). In the fecal occult blood test, the score of visible blood in stool was 4, and the rest of the stool was detected by the o-tolidine method. DAI scoring criteria are shown in Table1 below:

**TABLE 1:** The scoring standard of the DAI index.

Scores	Weight loss percentage	Fecal traits	Blood in the stool
0	No weight loss	Normal	No color change within 2 min
1	0%–5%	Softener stool	The color turns from light green to blue after 10 s of adding the reagent
2	5%–10%	Feces are shapeless and adhere to the anus	The color becomes blue and gradually changes to blue-brown
3	10%–15%	Loose stools and adhere to the anus	The color becomes blue-brown and gradually changes to blue-black
4	>15%	Severe diarrhea and soiled tail roots	The color becomes dark brown and gradually changes to brown-black

### 2.7 Histopathological analysis and mucus staining of colon

Large intestines were collected, photographed, and measured for length after sacrificing the mice. Colons without the cecum were washed with sterile saline to remove feces and stored in a tube for further experiments. About 1 cm of middle colon tissue was cut off and fixed with 4% paraformaldehyde. The fixed colonic tissues were dehydrated, embedded, and sectioned. Hematoxylin-eosin (HE), alcian blue (AB), and periodic acid-Schiff (PAS) staining were employed, respectively, to monitor the morphological changes among the five groups. The staining results will be photographed using a light microscope (Olympus, Japan). The score for the pathology of mice colon tissue was made by referring to several kinds of literature (Wu et al., 2020; Pan et al., 2022; Xiong et al., 2022), as detailed in Table 2.

**TABLE 2:** The scoring standard of histopathological analysis.

Scores	The morphology of colon tissue
0	Normal
1	Mild damage to the crypt, slight inflammatory cell infiltration
2	Moderate damage to the crypt, partial inflammatory cell infiltration
3	Severe damage to the crypt, massive inflammatory cells infiltrating, intact epithelium
4	Severe damage to the crypt, massive inflammatory cells infiltrating, epithelial loss

### 2.8 Detection of biochemical indicators of colonic tissue

Colonic tissues were homogenized on ice, and the protein concentration of the homogenate of each sample was determined using the Komas Brilliant Blue method after centrifuging at 10,000 g for 10 min. The level of CAT, GSH, MDA, and SOD was quantified by commercial kits to determine the degree of oxidative damage of colonic tissues in each group.

### 2.9 Quantitative real-time polymerase chain reaction (qRT-PCR)

QRT-PCR detected TLR4/MyD88/NF-κB pathway-related genes transcription in mice colonic tissues. Colonic tissue was cut up into small pieces with scissors and homogenized thoroughly by an electric homogenizer. RNA was extracted from colonic tissue with Trizol reagent. The purity and concentration of the extracted RNA were determined using NanoDrop 2000 (Thermo Fisher Scientific, United States). The RNA was reverse transcribed to cDNA using a reverse transcription kit (Vazyme, China). Then qRT-PCR was performed according to the instructions of AceQ Universal SYBR qPCR Master Mix (Vazyme, China). qRT-PCR reaction procedures were set as one cycle at 95°C for 5 min and 40 cycles at 95°C for 10 s and 60°C for 30 sec. Calculations were performed using the 2^−ΔΔCT^ method and β-actin as an internal reference. The primer sequences of the relevant genes are shown in Table 3.

**TABLE 3:** Primer sequences for quantitative real-time PCR reactions.

Gene name	Primer sequence
β-actin	Forward 5′-GCAGGAGTACGATGAGTCCG-3′
	Reverse 5′-ACGCAGCTCAGTAACAGTCC-3′
TLR4	Forward 5′-ATG​GCA​TGG​CTT​ACA​CCA​CC-3′
	Reverse 5′-GAG​GCC​AAT​TTT​GTC​TCC​ACA-3′
MyD88	Forward 5′- TCA​TGT​TCT​CCA​TAC​CCT​TGG​T-3′
	Reverse 5′-AAA​CTG​CGA​GTG​GGG​TCA​G -3′
NF-κB p65	Forward 5′- ATGATCGCCACCGGATTGAA -3′
	Reverse 5′-AAGGACTTCCTTACCTGGCTTG-3′

### 2.10 Western blot analysis

PBS buffer containing PMSF and phosphatase inhibitor was added to colonic tissue to extract total proteins, and the Komas Brilliant Blue method was used to determine the protein content of supernatant after the colonic tissue was homogenized on ice and centrifuged at 12,000 g for 10 min. The extracted proteins were mixed by loading buffer and boiled for 10 min to denaturation. Extracted proteins were separated by sodium dodecyl sulfate-polyacrylamide electrophoresis (SDS-PAGE) on a 10% polyacrylamide gel. The proteins in the gel were then transferred to a nitrocellulose (NC) membrane, and then the membrane was blocked with 5% skimmed milk for 1.5 h. After incubation of the primary antibody overnight at 4°C, the membrane was washed three times with TBST. After incubating with horseradish peroxidase (HRP) labeled secondary antibody for 1 h at room temperature, the membrane was washed three times with TBST again for further exposure. Finally, protein visualization analysis was performed by the Enhanced chemiluminescence (ECL) system, and the results were quantified using ImageJ software.

### 2.11 Gut flora analysis

Colon tissues were rinsed with sterile saline, and intestinal contents were collected in sterilized EP tubes. Based on several results, such as colon length, weight, and tissue staining, we found that the TCEA-L group significantly affected DSS-induced UC. Therefore we chose the TCEA-L group for the subsequent intestinal flora analysis. Six samples in control, DSS, and TCEA-L groups were randomly selected for the next step of the intestinal flora study. After extracting each sample’s DNA, the DNA’s purity and concentration were analyzed by agarose gel electrophoresis. The V3-V4 variable region was amplified through PCR technology using diluted DNA as a template. PCR products were purified and normalized to equal concentrations after they qualified. NovaSeq 6000 was employed for sequencing after library construction. The original tags data was obtained after splicing the reads of each sample using FLASH, and the quality control of original tags data was finally carried out to gain effective tags. The effective tags of all samples were clustered by the Uparse algorithm, with 97% of the consistency will become the operational taxonomic units (OTU). The Venn diagram, sample richness and diversity, phylum and genus level difference, principal component, LEfSe, and function prediction among the three groups were then analyzed by Qiime, R, LEfSe, and other software. The whole service was done by Metware Biotech (Wuhan, Hubei, China).

### 2.12 Statistical analysis

Data are shown as means ± SD from three to eight replicate experiments using different mice. Real-time PCR data represent the mean ± SE for triplicate measurements on each tissue, with tissue sets isolated from three mice. Experimental data were analyzed using SPSS 26.0 (Chicago, IL, United States) software. Statistical differences between multiple groups were analyzed via one-way analysis of variance (ANOVA) followed by the least significant difference (LSD) test for comparisons. *p*-values less than 0.05 were considered statistically significant differences.

## 3 Results

### 3.1 Chemical composition of *T. chebula*

**FIGURE 1:**
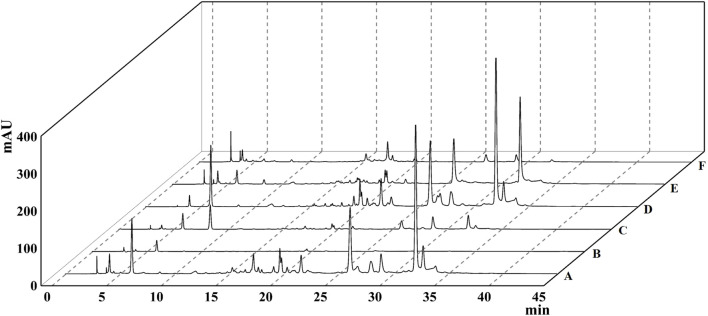
The HPLC chromatogram of different extracts of *Terminalia chebula*. **(A)** 95% ethanol extract. **(B)** Petroleum ether extract. **(C)** Dichloromethane extract. **(D)** Ethyl acetate extract. **(E)** n-Butanol extract. **(F)** Aqueous phase extract.

**FIGURE 2:**
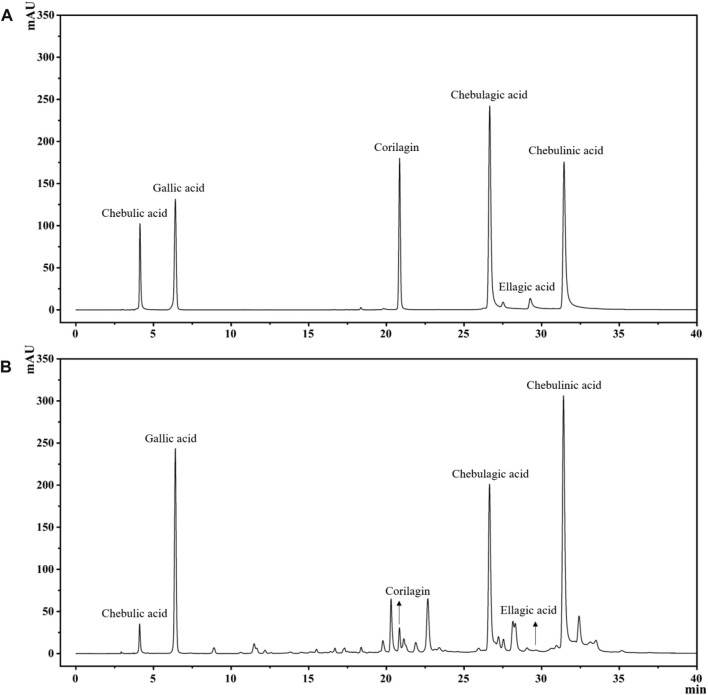
The HPLC chromatogram of TCEA and its main components, including Chebulic acid, Gallic acid, Corilagin, Chebulagic acid, Ellagic acid, and Chebulinic acid. **(A)** Standards. **(B)** TCEA Sample.

The extraction rate of *T. chebula* powder by ultrasonic extraction was 42.55%. After extraction with different solvents, the ethyl acetate fraction showed the highest extraction rate, and the petroleum ether fraction showed the lowest extraction rate, 44.479%, and 0.093%, respectively. The remaining parts included dichloromethane, n-butanol, and aqueous fractions, extracted at 0.640%, 15.882%, and 24.628%, respectively. By comparing the HPLC profiles of the five fractions, we observed that the components in petroleum ether, dichloromethane, and aqueous phase were relatively few. Still, the components mostly clustered in the ethyl acetate fraction compared with other fractions, as shown in Figure 1. To find out which main components are contained in the ethyl acetate extract, we performed qualitative analysis by standard compounds. It can be seen from Figure 2 that the ethyl acetate fraction contains chebulic acid, gallic acid, corilagin, chebulagic acid, ellagic acid, and chebulinic acid, where chebulinic acid, gallic acid, and chebulagic acid showed relatively high abundance under a UV absorption wavelength of 275 nm.

### 3.2 TCEA improves physiological index in DSS-induced UC mice

**FIGURE 3:**
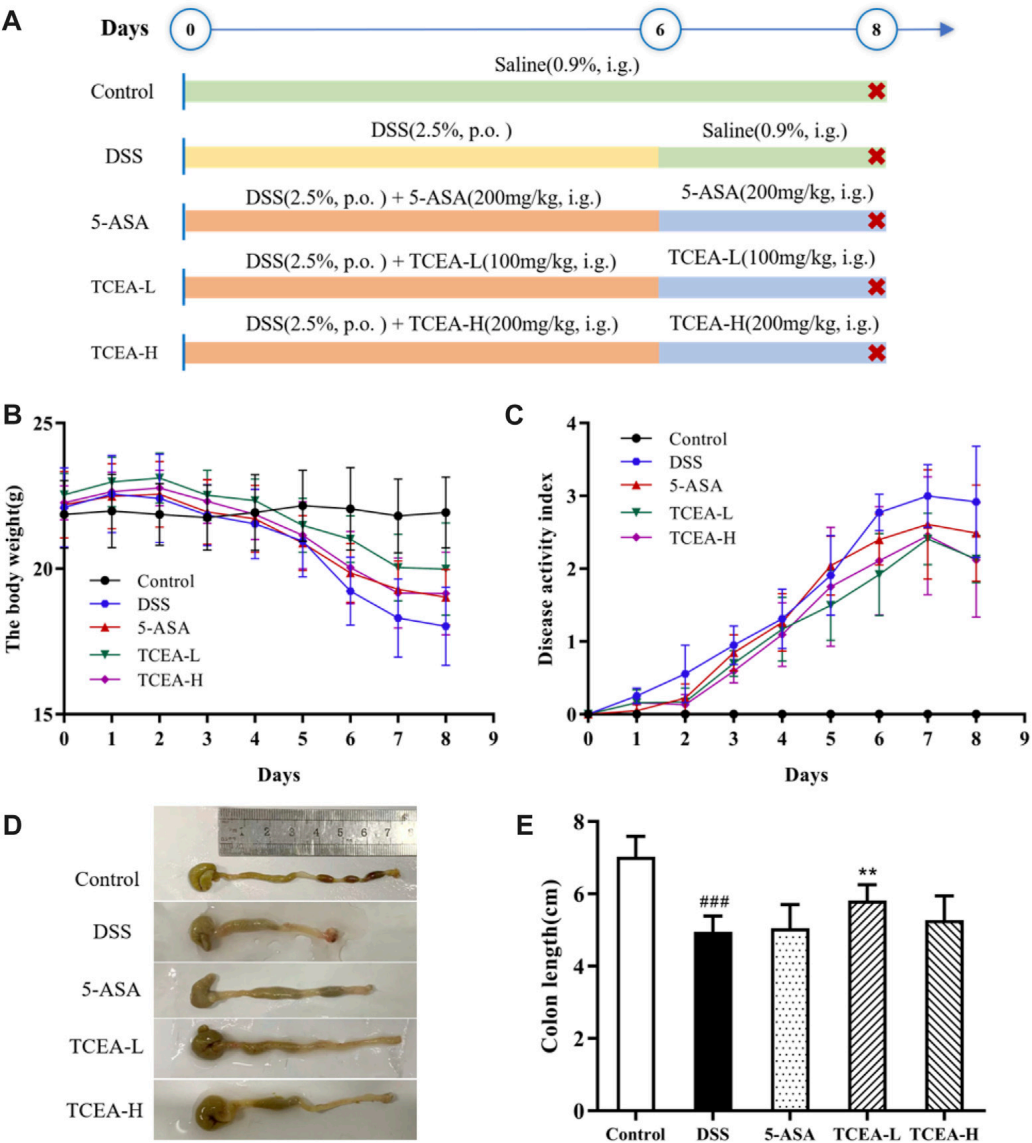
Effect of TCEA on the symptoms of DSS-induced colitis in mice. **(A)** Schematic diagram of animal experiment design. **(B)** The weight change of mice in each group during the experiment. **(C)** DAI index of colitis mice. **(D)** Pictures of the colon in different groups of mice. **(E)** Colonic length in different groups. ^#^ *p* < 0.05; ^##^ *p* < 0.01; ^###^ *p* < 0.001, compared with the control group. *p < 0.05; **p < 0.01; ***p < 0.001, compared with the DSS group.

After 3-day modeling, the feces of the DSS group began to be shapeless, and the DAI score increased significantly. After 7-day modeling, the mice in the DSS group were listless and appeared to have visible symptoms, including severe diarrhea and stool bleeding. At the same time, the DAI score reached its peak. Compared with the DSS group, mice in 5-ASA and TCEA-administered groups showed more activities and lower DAI scores. Meanwhile, the body weight of the mice in the DSS group decreased significantly throughout the experiment, with the highest rate of weight loss reaching 28.93%. 5-ASA and TCEA administration groups also had different degrees of body weight loss, but it was better than the DSS group (Figures 3B, C). Colon shortening is an essential feature of ulcerative colitis. Our results indicated that the colon length of the control group is the longest, with a reduction of more than 25% in the DSS group compared to the control group.5-ASA and different doses of TCEA treatments could ameliorate the colon shortening caused by DSS modeling (Figure 3D).

### 3.3 TCEA attenuates colonic injury in UC mice

**FIGURE 4:**
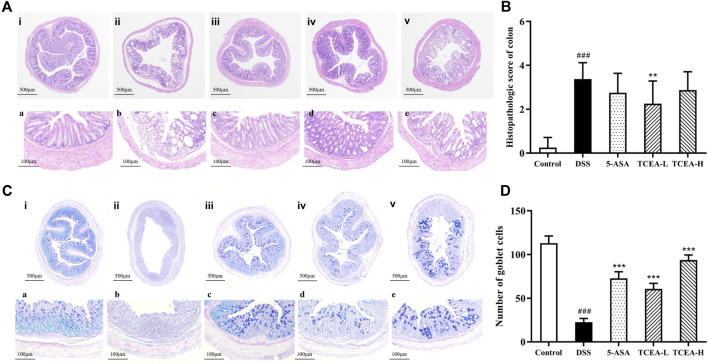
Effect of TCEA on histopathological damage in mice colons. **(A)** H&E staining of colons. **(B)** Histological score of colon tissues. **(C)** AB-PAS staining of colons. **(D)** Goblet cell count in colon tissue. (i, a) Control. (ii, b) DSS. (iii, c) 5-ASA. (iv, d) TCEA-L. (v, e) TCEA-H. Upper case letters represent ×40magnification, and lower case letters represent ×200magnification. ^#^ *p* < 0.05; ^##^ *p* < 0.01; ^###^ *p* < 0.001, compared with the control group. *p < 0.05; **p < 0.01; ***p < 0.001, compared with the DSS group.

HE staining results can be observed from Figure 4A, which demonstrated that the colonic mucosal, submucosal, and muscle layers were intact, the crypt surface was regular, the crypt was neatly arranged, and goblet cells were abundant in the control group mice. On the contrary, significant damage to the mucosal layer of colonic tissue, obvious destruction of glands and crypt, goblet cell loss, and inflammatory cell infiltration were observed in DSS group mice. According to the colon histopathological score (Figure 4B), it was found that the score of the DSS group was more than ten times higher than that of the control group, presenting an obvious upward trend, while the scores of the positive drug group and the TCEA treatment group decreased to a certain extent, among which the downward trend of TCEA-L was more evident. Mucus from goblet cells plays a vital role in intestinal protection. From the results of AB-PAS staining, we could see that the colon of the control group was not only full of mucus but also rich in goblet cells. After DSS induction, both mucus secretion and goblet cells were significantly reduced. The intervention of 5-ASA and TCEA alleviated the colon injury caused by DSS, and the number of goblet cells increased significantly with partial recovery of mucus secretion (Figures 4C, D).

### 3.4 TCEA affects oxidative stress parameters in the colon of UC mice

**FIGURE 5:**
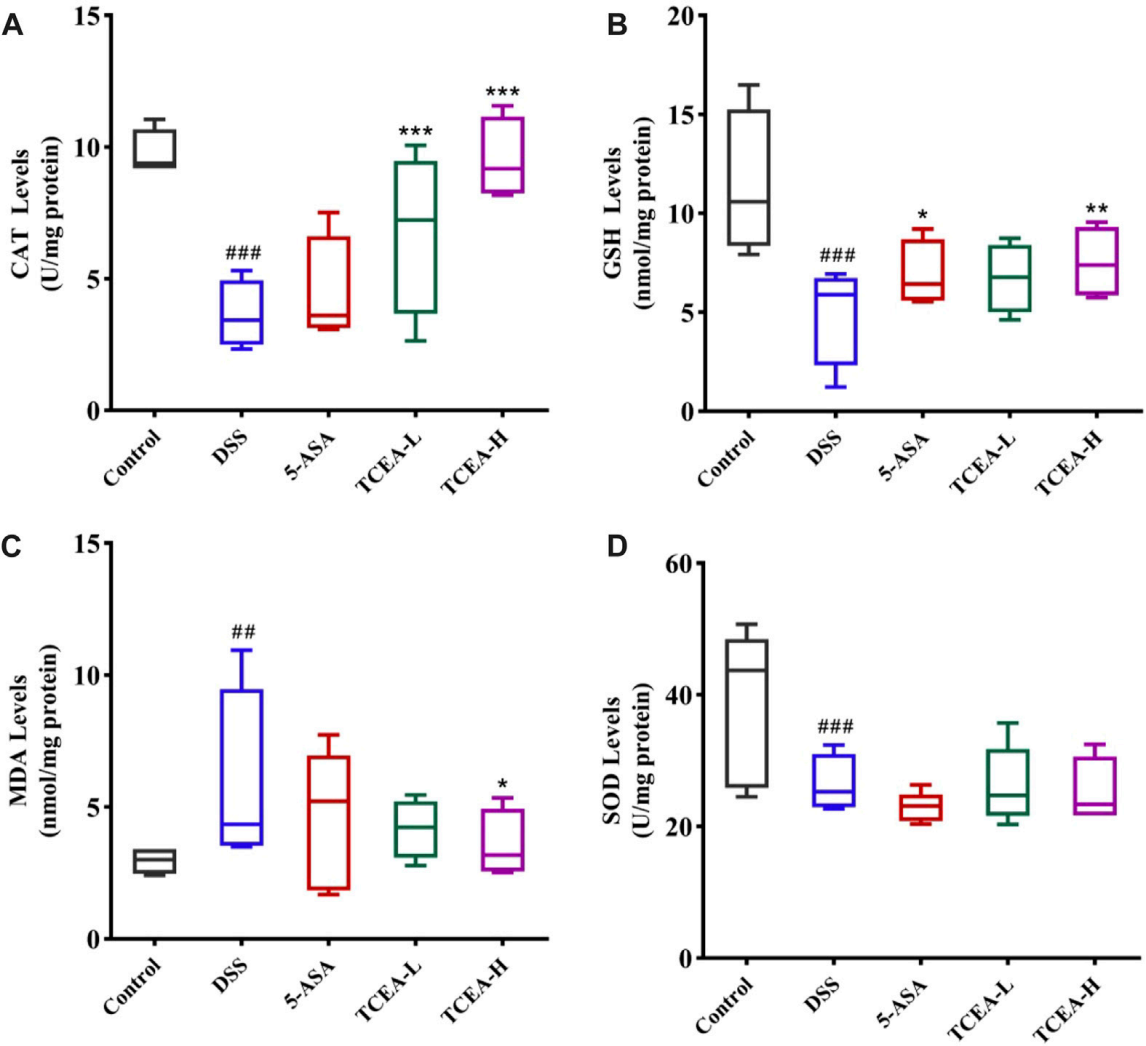
Effect of TCEA on parameters related to oxidative stress in the tissues of mice with DSS-induced colitis. **(A)** CAT levels. **(B)** GSH levels. **(C)** MDA levels. **(D)** SOD levels. ^#^ *p* < 0.05; ^##^ *p* < 0.01; ^###^ *p* < 0.001, compared with the control group. *p < 0.05; **p < 0.01; ***p < 0.001, compared with the DSS group.

Colonic tissue is associated with oxidative stress indicators of results, as shown in Figure 5. The contents of CAT, GSH, and SOD in the DSS group were significantly decreased, while the level of MDA was specifically reflected in the sharp increase in the DSS group. These changes were reversed after 5-ASA or TCEA treatment. Our results confirmed that TCEA increases the levels of CAT and GSH (Figures 5A, B) and inhibits MDA production in the colonic tissue (Figure 5C). Besides, although TCEA intervention reduced SOD levels to some extent, no significant difference was detected (Figure 5D). Overall, TCEA administration could relieve on oxidative stress, in which TCEA-H group was more effective.

### 3.5 Effect of TCEA on TLRA/MyD88/NF-κB pathway in UC mice

**FIGURE 6:**
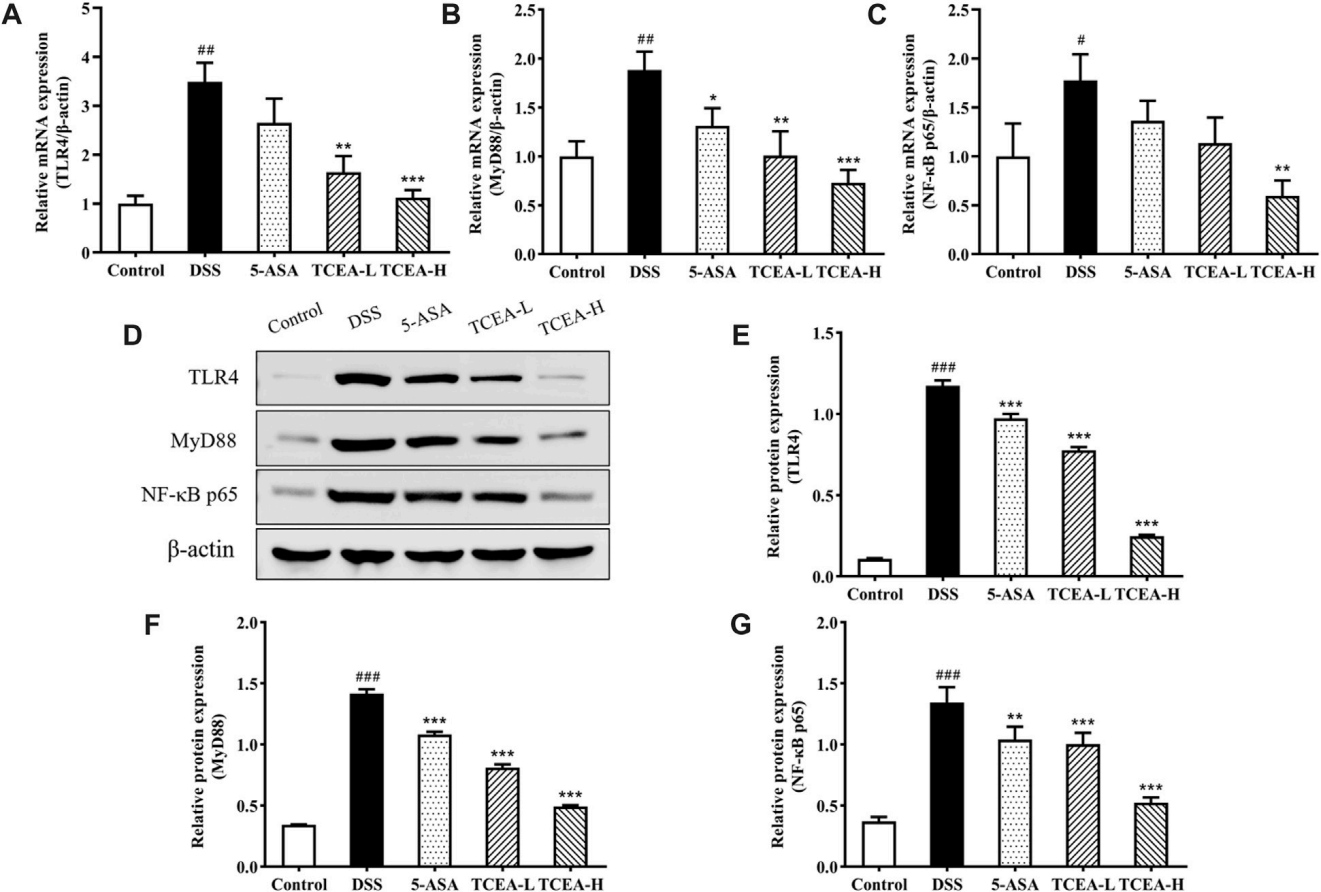
The protein and mRNA expression of TLR4, MyD88, and NF-κB p65 in colonic tissues of various groups of mice. **(A)** TLR4 mRNA expression **(B)** MyD88 mRNA expression. **(C)** NF-κB p65 mRNA expression. **(D)** Representative Western blotting images for TLR4/MyD88/NF-κB signal pathway. **(E)** TLR4 protein expression. **(F)** MyD88 protein expression. **(G)** NF-κB p65 protein expression. ^#^ *p* < 0.05; ^##^ *p* < 0.01; ^###^ *p* < 0.001, compared with the control group. *p < 0.05; **p < 0.01; ***p < 0.001, compared with the DSS group.

TLR4/NF-κB signaling pathway is one of the vital signaling pathways mediating inflammation and plays a key role in the occurrence and development of UC (Wu et al., 2020). As shown in Figure 6, we determined the mRNA expression levels of TLR4, MyD88, and NF-κB p65 by qRT-PCR to detect whether the mechanism of action of TCEA’s anti-inflammatory effect involved in TLR4/NF-κB signaling pathway. Our results showed apparent increases in the mRNA expressions of TLR4, MyD88, and NF-κB p65 after DSS induction. Nevertheless, after different doses of TCEA intervention, the above genes’ expressions were reversed to the control level, especially the TCEA-H group, which showed the best reverse effect (Figures 6A–C). We also validate the expression of TLR4, MyD88, and NF-κB p65 at the protein level. Western blot showed that the expression of TLR4, MyD88, and NF-κB p65 in colon tissue of the DSS group increased sharply, and their expressions were gradually inhibited in 5-ASA, TCEA-L, and TCEA-H groups compared with those of DSS group (Figures 6D–G).

### 3.6 TCEA alters the abundance and diversity of intestinal flora in DSS-induced UC mice

**FIGURE 7:**
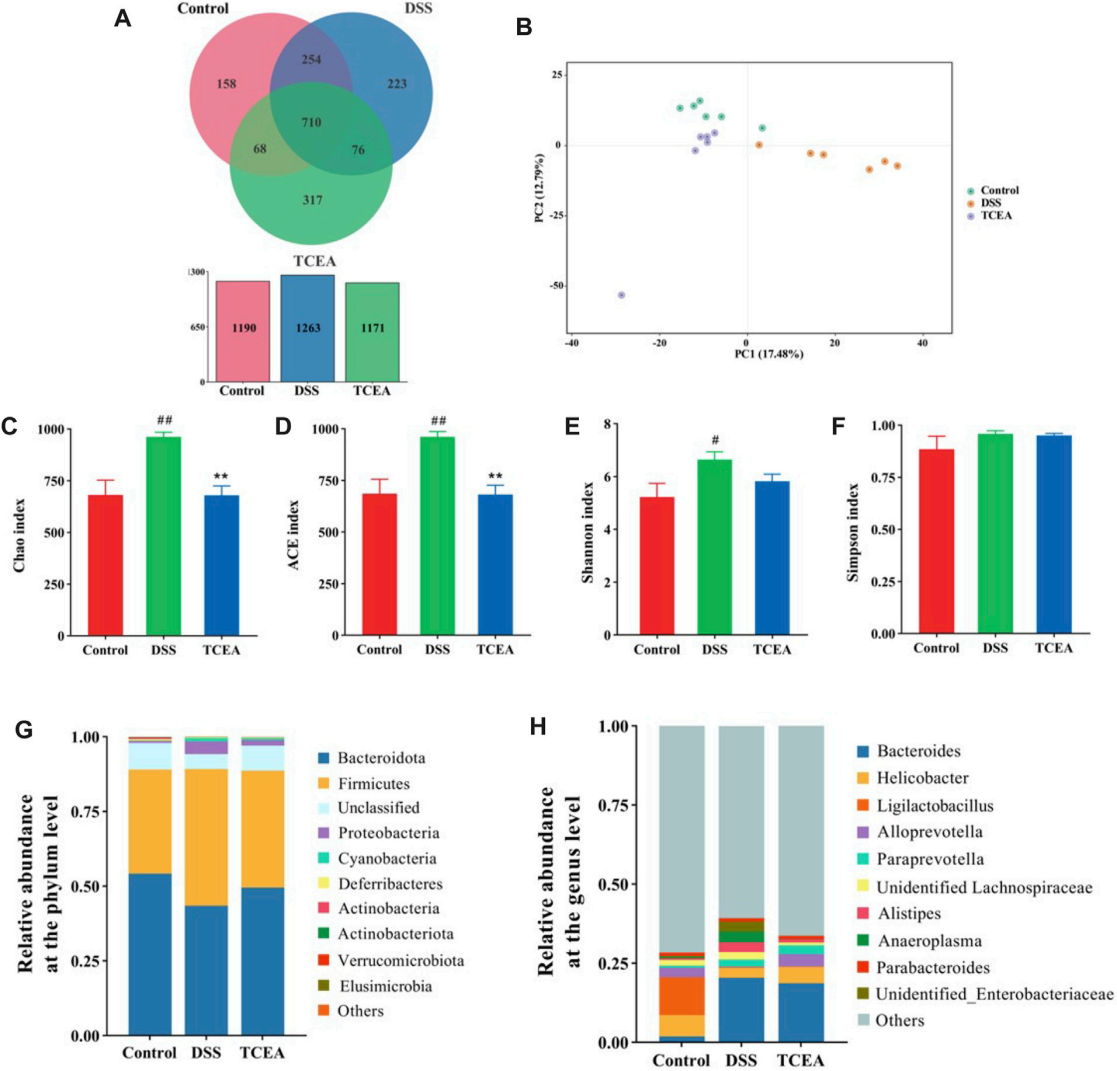
Effect of TCEA on the intestinal microflora of mice with DSS-induced UC. **(A)** Venn diagram of species in control, DSS, and TCEA-L group. **(B)** PCA analysis of three groups. **(C)** Chao index. **(D)** ACE index. **(E)** Shannon index. **(F)** Simpson index. **(G)** Relative abundance of species at the phylum level for three groups of gut flora. **(H)** Relative abundance of species at the genus level for three groups of gut flora. ^#^ *p* < 0.05; ^##^ *p* < 0.01; ^###^ *p* < 0.001, compared with the control group. *p < 0.05; **p < 0.01; ***p < 0.001, compared with the DSS group.

It was found that intestinal flora plays a non-negligible role in the occurrence of UC, so we exploited the 16S sequencing technique to test whether TCEA alters the intestinal flora. The Venn diagram demonstrated the changes in the number of intestinal microbes among three groups; the OTU numbers of intestinal flora in control, DSS, and TCEA-treated groups were 1190, 1263, and 1171, respectively (Figure 7A), which indicated that compared with the control group, DSS treatment increased the total number of intestinal microbes in the DSS group, while TCEA treatment inhibited the trend. In addition, the principal component analysis (PCA) results on the OTU level suggested a clear divergence in the microbial community composition between the control, DSS, and TCEA treatment groups. Still, the composition was more similar between the control and TCEA treatment groups (Figure 7B). α-diversity can reflect the richness and diversity of the sample microbial communities, where Chao1 and ACE indices reflect the richness and Shannon and Simpson’s index reflects diversity. These four indexes in the DSS group all showed an uptrend. Still, the Chao1 and ACE indexes were decreased after TCEA treatment, indicating that the intestinal flora richness of mice was significantly reduced (Figures 7C, D). However, Shannon and Simpson’s index had no significant difference after TCEA treatment showing the diversity changed weakly (Figures 7E, F).

### 3.7 TCEA alters the composition of intestinal flora in DSS-induced UC mice

To clarify which bacteria make the discrepancy between the different groups, the composition of the microbial community was contrasted at the phylum and genus levels. At the phylum level, the composition of the flora was dominated by the *Bacteroidota* and *Firmicutes*, accounting for more than 75% of the species’ abundance. Compared with the control group, the DSS-induced group showed a decrease in the relative abundance of the *Bacteroidota* and an increase in the relative abundance of the *Firmicutes*, but TCEA treatment suppressed these changes. Meanwhile, the relative abundance of *Proteobacteria* in the DSS and control group assumed bulky diversity (Figure 7G). Figure 7H demonstrates the differences in microbial communities at the genus level between the control, DSS, and TCEA -treated groups; we can see that *Bacteroides*, *Alistipes,* and *Anaeroplasma* grown evidently in the DSS group, while *Helicobacter* and *Alloprevotella* presented a downward trend. TCEA treatment reversed this phenomenon by increasing the relative abundances of *Helicobacter* and *Alloprevotella* and reducing the relative lots of *Bacteroides*, *Alistipes*, and *Anaeroplasma*.

**FIGURE 8:**
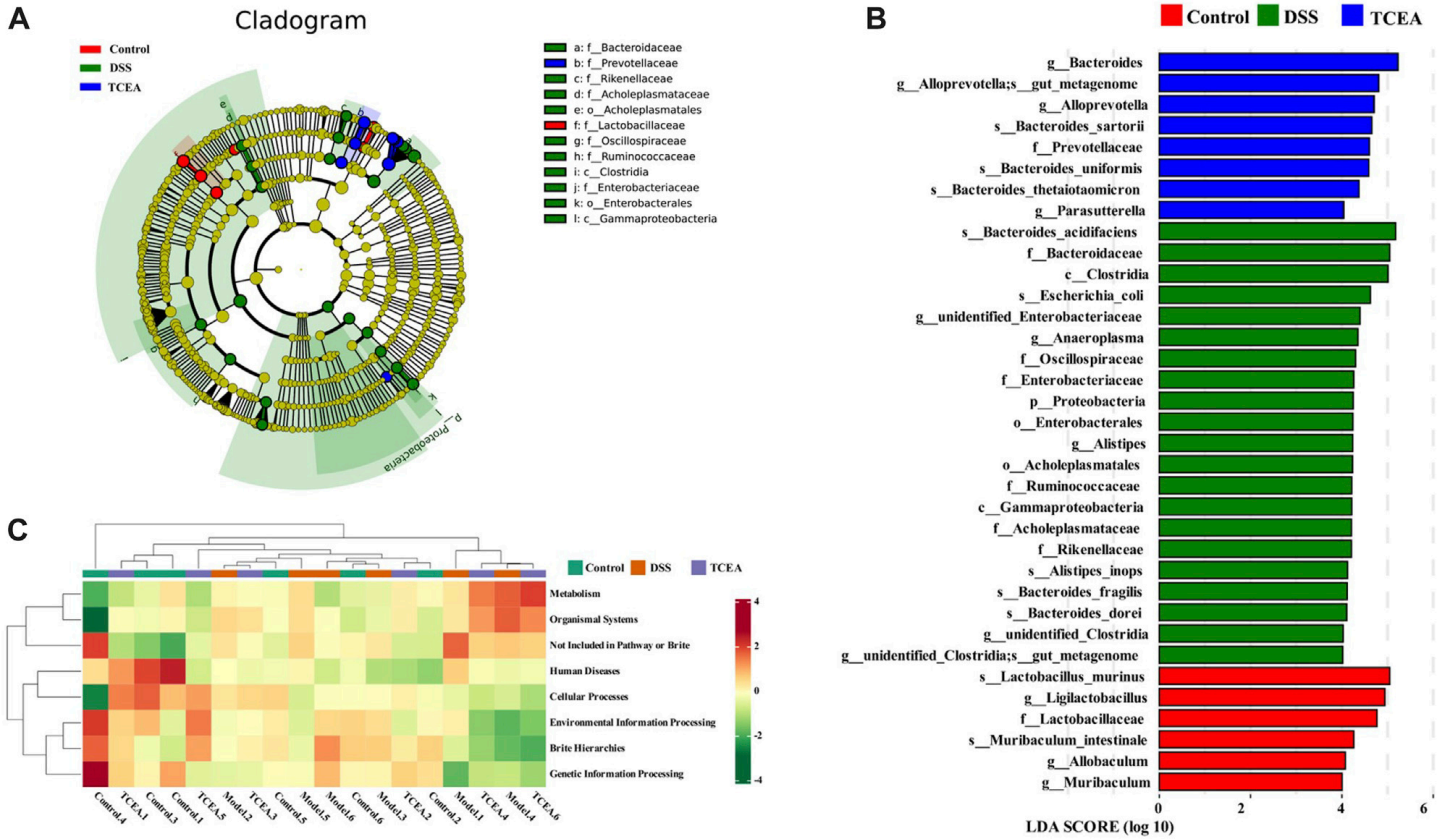
Analysis of the variability of intestinal flora in three groups and prediction of flora function. **(A)** Evolutionary branching diagram based on OTU. **(B)** Differences in the microbial taxa shown by LEfSe analysis. **(C)** Heat map of PICRUSt2 predicting the function of each sample in control, DSS, and TCEA-L groups.

LEfSe analysis was used further to explore the bacteria with significant differences among the groups. A total of 35 bacteria showed obvious changes among the control, DSS, and TCEA treatment groups, and the evolutionary branching diagram shows the major microorganisms in each group, with the diameter size of the circles on each stratum proportional to the relative abundance size (Figures 8A, B). Functional prediction based on PICRUSt2 function for each sample population revealed that the control group was more positively correlated with genetic information processing, environmental information processing, and human disease but negatively correlated with metabolic and organismal systems. However, the DSS group showed the opposite correlation compared to the control group, while the TCEA treatment group showed a similar correlation to the control group (Figure 8C).

**FIGURE 9:**
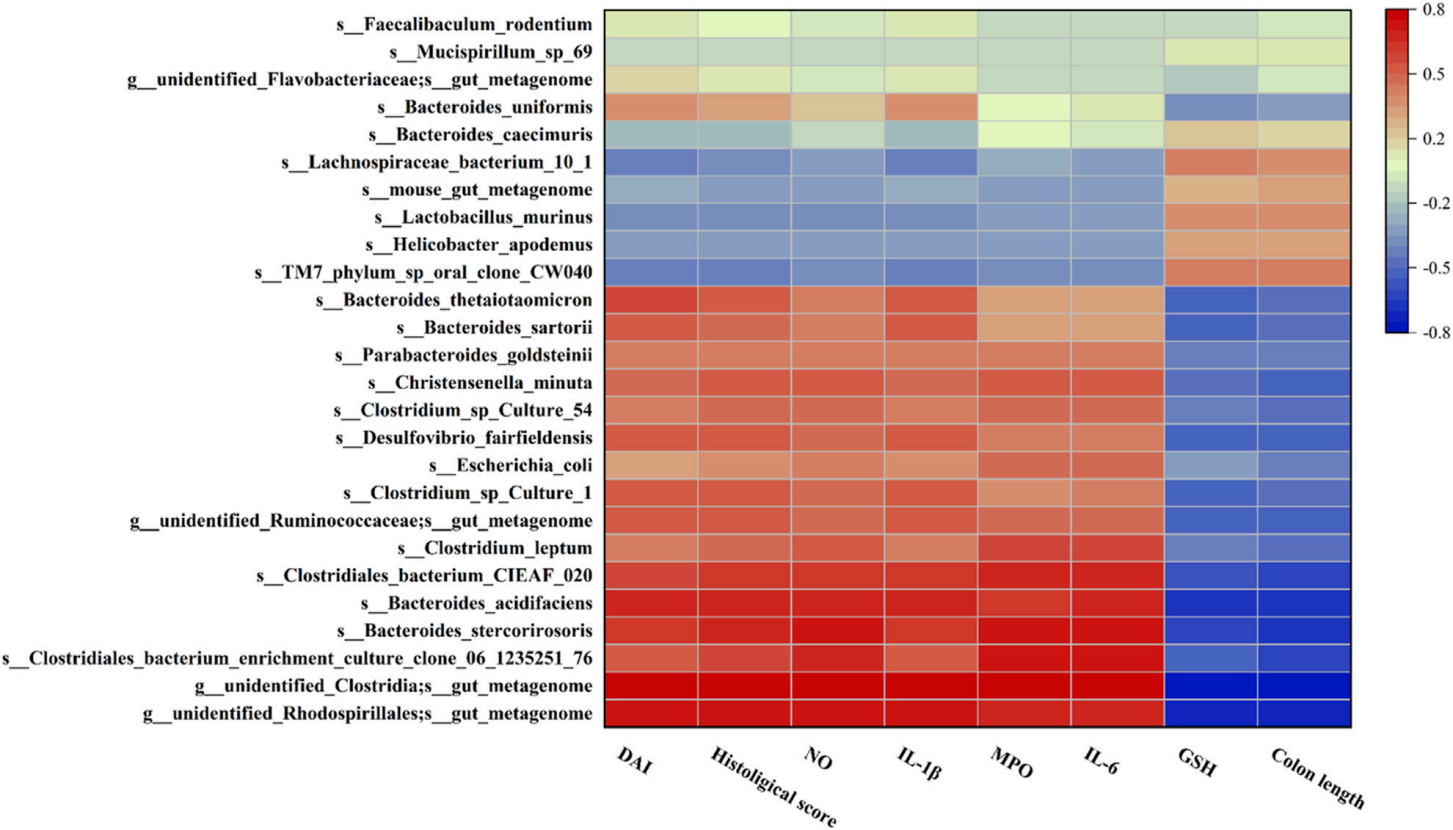
Heatmap of Pearson correlation analysis between the changes in the gut microbiota and UC parameters. Red and blue represent positive and negative correlations, respectively.

To discuss whether changes in the gut microbiota in mice are associated with UC-related biomarkers, Pearson correlation analysis was used to analyze microbiota at species level with DAI, colon length, histological score, GSH, and inflammatory factor levels (Figure 9). These results indicated that *Bacteroides_stercorirosoris*, *Bacteroides_acidifaciens*, *Clostridiales_bacterium_CIEAF_020* were negatively correlated with GSH and colon length. However, these three bacteria showed a significant positive correlation with other parameters. Moreover, *Desulfovibrio_fairfieldensis*, *Bacteroides_sartorii*, and *Bacteroides_thetaiotaomicron* exhibited an obvious positive correlation with IL-1β, while *Clostridium_leptum* and *Christensenella_minuta* exhibited an obvious positive correlation with IL-6. The above evidence suggests the nonnegligible role of intestinal flora in the remission of UC.

## 4 Discussion

UC is a chronic inflammatory bowel disease with a long and recurrent course. It is reported that the incidence of UC is on the rise in both developed and developing countries, and UC may occur at any age (Feuerstein et al., 2019). The available therapeutic drugs for UC include aminosalicylic acid, corticosteroids, and immunomodulators, but the use of these drugs may cause gastrointestinal reactions, osteoporosis, moon face, and other adverse reactions (Kobayashi et al., 2020; Raine et al., 2022). Therefore, finding safer and more effective drugs to relieve UC is particularly requisite. Nowadays, quantities of evidence confirm that TCM has an important influence on treating UC. Researchers found that a variety of Chinese medicinal herbs, including “*Platycodon grandiflorum*,” “*Scutellaria baicalensis*,” and “*Flos lonicera*,” have great potential for relieving UC (Zheng et al., 2022). In this study, the extract of *T. chebula* was also proved to be effectual against UC induced by DSS.

We characterized the six major compounds in TCEA by HPLC technique, including Chebulic acid, Gallic acid, Corilagin, Chebulagic acid, Ellagic acid, and Chebulinic acid. It was reported that gallic acid and ellagic acid were proven to have anti-inflammatory effects inhibiting LPS-induced NO, IL-6, and PGE-2 production in RAW264.7 cells (BenSaad et al., 2017). Chebulinic acid, another active ingredient in TCEA, was also proved to have an excellent anti-inflammatory effect, which significantly improved foot swelling and reduced the mean joint index and pathology score in collagen-induced arthritis mice (Lu et al., 2020). Therefore, we speculate that these constituents may contribute to the anti-inflammatory activity of *T. chebula*.

The symptoms and histological changes of DSS-induced UC are very similar to those of human UC, making it an ideal model for studying the pathogenesis of UC and evaluating drug efficacy (Okayasu et al., 1990). DSS can inhibit epithelial cell proliferation, destroy the intestinal mucosal barrier, cause inflammation, and lead to colitis (Wirtz et al., 2017). Thus, we established a mice model of acute UC using 2.5% DSS and further evaluated the effect of TCEA on DSS-induced acute UC in mice. Mice with sustained DSS ingestion will experience significant weight loss, diarrhea, and stool blooding with severe colon shortening. Our results found that TCEA intervention reduced the DAI index, improved diarrhea and blood in stool, and inhibited colon shortening caused by DSS in the model group of mice. Pathological results displayed that DSS modeling could damage the crypt structure of colon tissue, leading to inflammatory cell infiltration (Dai et al., 2023). The goblet cells’ primary functions include synthesizing and protective secretion of mucus, sensing changes in the local environment, participating in intestinal immunity, and playing a critical role in regulating intestinal health (Gustafsson and Johansson, 2022). Loss of goblet cells and a decrease in mucus secretion were also observed in colon tissue slices of the DSS group of mice. TCEA intervention can reverse these DSS-induced changes and inhibit oxidative stress in mice. The occurrence of UC is also affected by changes in the body’s oxidative stress level (Bourgonje et al., 2020). Zhou et al. confirmed that the ethyl acetate fraction of Maqui berry extract could contribute to the effects of UC by influencing the oxidative stress level in UC mice (Zhou et al., 2019). Our results also proved that TCEA affected oxidative stress levels mainly by affecting the levels of oxidative products, antioxidants, and antioxidant enzymes.

The inflammation process is daedal, which is influenced and regulated by multiple factors, such as inflammatory triggers and mediators (Medzhitov, 2010). When the body is exposed to external stimuli, the innate immune system initiates the inflammatory response by activating pattern recognition receptors (Yang et al., 2020). As an important receptor for innate immune recognition, TLR4 is indispensable in homeostasis, intestinal inflammation, and inflammatory bowel disease *in vivo* (Garcia et al., 2020; Dai et al., 2022). There is evidence that DSS modeling will lead to ascending expression of TLR4 in colonic tissue, which is consistent with our findings (Xiong et al., 2022). The expression of TLR4 increased obviously at both the transcriptional and translational levels. When the TLR4 receptor is activated, it activates the MyD88-dependent signal transduction pathway, activating NF-κB and producing pro-inflammatory mediators (Yang et al., 2021). NF-κB is a vital transcription factor that regulates immune response and plays a crucial role in the pathogenesis of UC (Xiong et al., 2022). Activation of NF-κB is related to the interaction of IκB; by phosphorylation, NF-κB p65 will release from the p65-p50-IκB complex, which leads to the production of cytokines such as IL-1β and promotes inflammation (Bian et al., 2022). Thus, we explored the regulatory function of TCEA on the TLR4/MyD88/NF-κB pathway in UC mice induced by acute DSS modeling. In this work, the experiment results proved that the expressions of MyD88 and NF-κB p65 at mRNA and protein levels were significantly increased by DSS administration. In contrast, the expressions of MyD88 and NF-κB p65 were obviously inhibited by TCEA treatment. It was found that methyl gallate and melatonin alleviated DSS and TNBS-induced UC by acting on TLR4/MyD88/NFκB signaling pathway (Chamanara et al., 2019; Zhou et al., 2022). Our experiment results displayed that TCEA, including methyl gallate, affects UC and may be related to the inhibition of the TLR4/MyD88/NF-κB signaling pathway.

Numerous and diverse microbiota inhabit the mucosal surface of the gut, on which many of our basic physiological and metabolic functions and proper immune protection depend (Ivanov and Honda, 2012). In recent years, growing evidence has supported the crucial role of intestinal flora in UC (Eom et al., 2018; Olaisen et al., 2019). Researchers constructing an immunodeficiency animal model by gene knockout technology found that mice showed no intestinal inflammation in a sterile environment, but colitis appeared after recovery of enteric flora (Wang et al., 2019). The relationship between intestinal flora and UC was explored because intestinal flora significantly differed between UC patients and healthy control groups, with *Tenericutes* and pathogenic intestinal bacteria significantly increasing. In contrast, *Synergistetes*, *Veillonella,* and other intestinal bacteria significantly decreased (Tang et al., 2021). A study found intestinal flora transplantation was valid in reducing inflammatory responses, correcting intestinal flora dysbiosis, and promoting restoration of intestinal barrier function in UC patients (Wang et al., 2023). Another study also demonstrated that intestinal flora transplantation could regulate DSS-induced intestinal flora dysbiosis in UC mice and reduce intestinal inflammation in mice (Li Y. Y. et al., 2022a; Li D. et al., 2022b). These evidences showed that changes in the intestinal flora are closely related to the development of UC. As expected, herbs can also influence UC by regulating intestinal flora. The herbal medicine *Pulsatilla chinensis* (Bunge) Regel can contribute to regulating the composition and diversity of intestinal flora in mice by enhancing the abundance of beneficial bacteria while decreasing the abundance of harmful bacteria, thereby curing UC (Liu et al., 2021). 16S rRNA sequencing is a widely used high-throughput sequencing technique for bacterial identification that enables rapid and comprehensive detection of microbial composition and diversity (Zou et al., 2020). In this study, we investigated the effect of TCEA on intestinal flora by 16S rRNA sequencing. Results showed differences in microflora composition among the control group, DSS group, and TCEA treatment group, but the composition of the control group and TCEA treatment group were similar. In addition, DSS administration increased the number of OTUs of intestinal flora and changes in flora richness and diversity in the DSS group. *Bacteroidota*, *Firmicutes*, *Proteobacteria*, and *Actinobacteria* are the four main phyla of the intestinal flora (Alam et al., 2020). And our results supported the similar conclusions that the *Bacteroidota*, *Firmicutes*, *Proteobacteria*, and *Actinobacteria* accounted for over 90% of the phylum level abundance. Meanwhile, TCEA effectively recovered the DSS-induced decrease in the relative abundance of *Bacteroidota* and the increase in the relative abundance of *Firmicutes*. Notably, colitis promotes a clear expansion of the *Proteobacteria* (Shin et al., 2015). Our data confirmed that DSS induction led to a more than 5-fold increase in the abundance of the *Proteobacteria* compared to the control and that TCEA treatment largely suppressed this trend. Besides, it is worth noting that the results of the prediction of gut flora function showed a positive correlation with metabolic and organismal systems in the DSS group, while the TCEA intervention reversed this change, with a final situation more similar to that of the control group. The above data suggested that TCEA may alleviate UC by acting on the intestinal flora composition and ameliorating intestinal flora dysfunction.

## 5 Conclusion

In conclusion, we examined the effects of TCEA on DSS-induced UC in mice. Our results suggested that TCEA exerted a protective influence on UC mainly by alleviating UC symptoms, ameliorating colonic tissue morphology, relieving oxidative stress *in vivo*, and inhibiting TLR4/MyD88/NF-κB pathway activation. At the same time, the regulation of intestinal microorganisms by TCEA has contributed to minimizing the inflammatory response in UC mice. The above results demonstrate that TCEA can be used as a novel anti-UC drug candidate.

## Data Availability

The datasets presented in this study can be found in online repositories. The names of the repository/repositories and accession number(s) can be found below: https://www.ncbi.nlm.nih.gov/, PRJNA975979.
